# Fractionated head and neck irradiation impacts taste progenitors, differentiated taste cells, and Wnt/β-catenin signaling in adult mice

**DOI:** 10.1038/s41598-019-54216-9

**Published:** 2019-11-29

**Authors:** Dany Gaillard, Lauren A. Shechtman, Sarah E. Millar, Linda A. Barlow

**Affiliations:** 1 Department of Cell & Developmental Biology, University of Colorado Anschutz Medical Campus, Mail Stop 8108, 12801 East 17th Avenue, Aurora, CO 80045 USA; 2 Rocky Mountain Taste & Smell Center, University of Colorado Anschutz Medical Campus, Mail Stop 8108, 12801 East 17th Avenue, Aurora, CO 80045 USA; 3 Black Family Stem Cell Institute, Icahn School of Medicine at Mount Sinai, New York, NY USA; 4 Department of Cell, Developmental and Regenerative Biology, Icahn School of Medicine at Mount Sinai, New York, NY USA; 5 Department of Dermatology, Icahn School of Medicine at Mount Sinai, New York, NY USA

**Keywords:** Regeneration, Cell proliferation, Differentiation, Disease model

## Abstract

Head and neck cancer patients receiving conventional repeated, low dose radiotherapy (fractionated IR) suffer from taste dysfunction that can persist for months and often years after treatment. To understand the mechanisms underlying functional taste loss, we established a fractionated IR mouse model to characterize how taste buds are affected. Following fractionated IR, we found as in our previous study using single dose IR, taste progenitor proliferation was reduced and progenitor cell number declined, leading to interruption in the supply of new taste receptor cells to taste buds. However, in contrast to a single dose of IR, we did not encounter increased progenitor cell death in response to fractionated IR. Instead, fractionated IR induced death of cells within taste buds. Overall, taste buds were smaller and fewer following fractionated IR, and contained fewer differentiated cells. In response to fractionated IR, expression of Wnt pathway genes, *Ctnnb1*, *Tcf7, Lef1* and *Lgr5* were reduced concomitantly with reduced progenitor proliferation. However, recovery of Wnt signaling post-IR lagged behind proliferative recovery. Overall, our data suggest carefully timed, local activation of Wnt/β-catenin signaling may mitigate radiation injury and/or speed recovery of taste cell renewal following fractionated IR.

## Introduction

Head and neck cancers represent ~4% of all cancers in the United States of America^1^. Treatments generally include surgery, chemotherapy and radiotherapy. Radiotherapy consists of small, fractionated X-ray doses of 1–2 Gy administered daily for up to 7 weeks^2^. Although new radiotherapy protocols^2^, including intensity-modulated radiation therapy (IMRT), volumetric modulated arc therapy (VMAT) and image-guided radiation therapy (IGRT), minimize exposure of healthy tissue, side effects remain common. Patients undergoing radiotherapy suffer from nausea, xerostomia (dry mouth), mucositis (oral blistering), difficulty swallowing, as well as taste disturbances including dysgeusia (distorted taste) and ageusia (taste loss)^3–6^. Radiation (IR)-induced taste dysfunction frequently causes depression due to lack of pleasure associated with eating, loss of appetite and motivation to eat, and weight loss from lower nutrient intake^7–12^. Thus, taste dysfunction is an important and distressing side effect for head and neck cancer patients^13,14^. Because the mechanisms underlying taste disturbance are poorly understood, targeted treatments to mitigate this serious consequence of radiotherapy have yet to be developed.

Under homeostasis, taste progenitor cells located outside of taste buds continually divide and supply new taste cells to each bud, thereby replacing the steady loss of aged cells^15,16^. We showed previously in mice that a single radiation dose administered to the head and neck induced a dramatic, albeit transient reduction in progenitor cell proliferation^17^, leading to interrupted supply of new taste cells, followed by a transient reduction in differentiated taste cells^17^. While this study was the first to comprehensively explore the impact of IR on taste bud cell renewal^17^, a single dose paradigm does not reflect conventional fractionated IR experienced by patients. In contrast to the rapid recovery of taste bud homeostasis as well as taste function in mice following a single dose^17,18^, patients can suffer from taste disturbance for months and even years following fractionated IR^14,19,20^. Here, we sought to establish how at a cellular level, taste homeostasis is affected by repeated IR.

The Wnt/β-catenin pathway controls the development and maintenance of multiple tissues, and activating mutations of the pathway are associated with the development of cancer^21^. Wnt ligands bind to coreceptors, triggering the release of β-catenin from a destruction complex, allowing its translocation to the nucleus where it binds LEF/TCF transcription factors to activate transcription of target genes^21^. Wnt/β-catenin signaling is also a major regulator of taste bud homeostasis; it is required for progenitor maintenance and proliferation, and functions in taste cell fate decisions^16,22–25^. Additionally, in a host of tissues, Wnt/β-catenin signaling functions in regeneration following injury, including IR damage^26–34^; thus here we investigated if and how Wnt signaling in taste epithelium is affected by fractionated IR.

## Results

### Fractionated IR results in reduced taste buds in posterior CVP and anterior FFP

**Figure 1:**
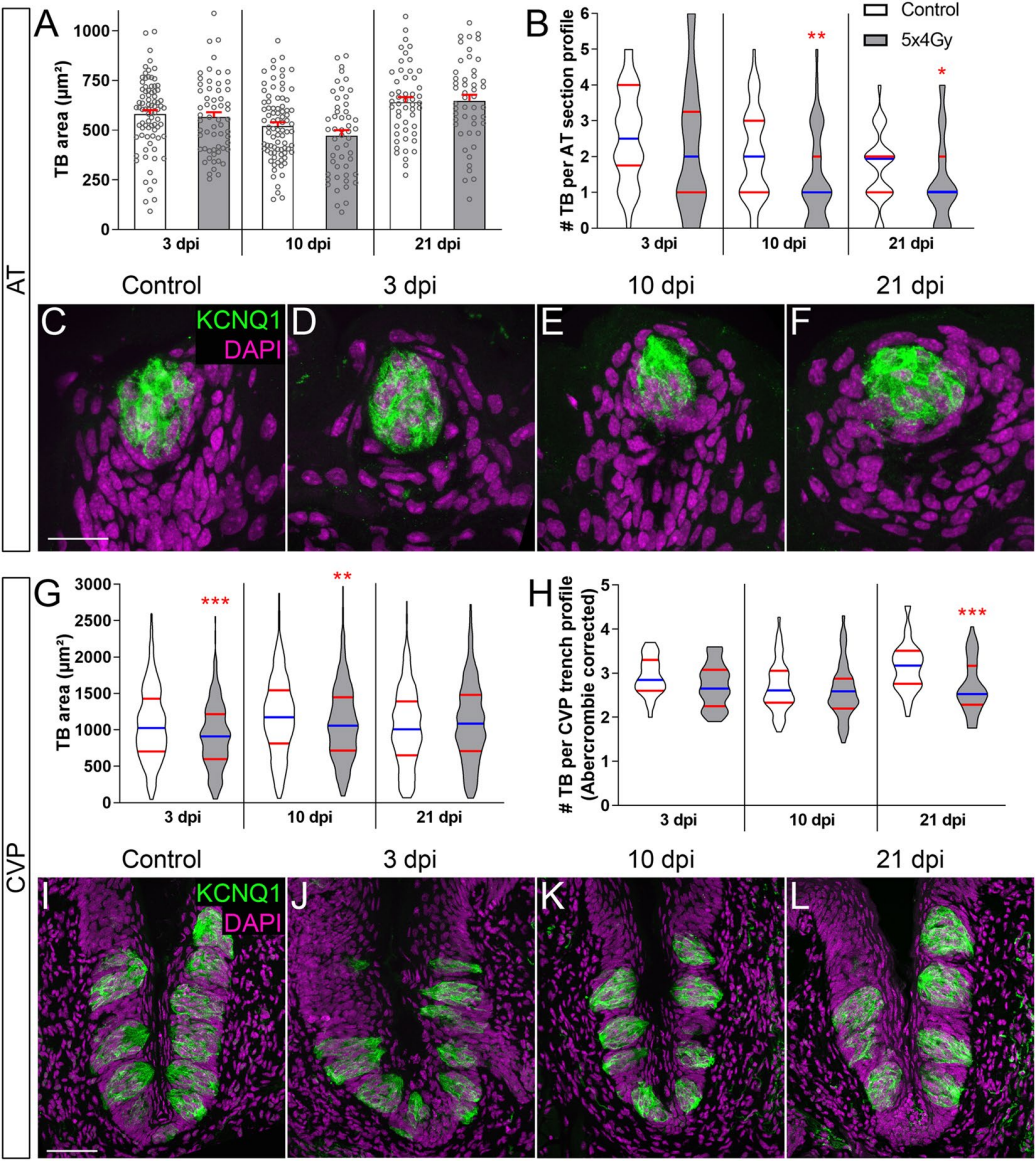
The number, but not size, of FFP taste buds is reduced following fractionated irradiation, while both size and numbers are diminished in the CVP. The area of KCNQ1^+^ FFP taste bud profiles (green) was used as a proxy for taste bud size, and was unaffected in irradiated mice compared to controls (**A**,**C**–**F**). The area of taste bud profiles was significantly smaller in irradiated CVP compared to controls at 3 and 10 dpi, and recovered at 21 dpi (**G**,**I**–**L**). The number of taste bud profiles remained unchanged in the CVP at 3 and 10 dpi, and was slightly reduced at 21 dpi (**H**,**I**–**L**). Fewer FFP taste buds were present in irradiated tongues at 10 and 21 dpi (**B**). Data are represented as violin plots with median (blue line) and 1^st^ and 3^rd^ quartile (red lines), or mean ± SEM. Mann & Whitney test in violin plots, Student’s t-test in mean ± SEM graph (*p < 0.05, **p < 0.01, ***p < 0.001). Taste bud Eqdiameter (see methods) in control FFP was not significantly different than irradiated FFP at any time points (Mann & Whitney test, Supplementary Fig. S1A) but taste bud Eqdiameter was significantly smaller in irradiated CVP taste buds at 3 and 10 dpi (Mann & Whitney test, Supplementary Fig. S1B); therefore, Abercrombie correction was applied to counts of CVP but not FFP taste buds (uncorrected data shown in Supplementary Fig. S1C). Representative pictures are compressed confocal z-stacks. Scale bars 20 µm in (**C)**, applies to (**D**–**F**), 50 µm in (**I)**, applies to (**J**–**L)**. FFP Control vs IR mice 3 dpi, N = 4 vs 3; 10 dpi, N = 4 vs 4; 21 dpi N = 3 vs 4. CVP Control vs IR mice 3 dpi, N = 4 vs 3; 10 dpi, N = 6 vs 6; 21 dpi N = 6 vs 7. (**A**) Control vs IR FFP taste bud profiles 3 dpi, n = 81 vs 59; 10 dpi, n = 83 vs 51; 21 dpi, n = 51 vs 49. (**B**) Control vs IR FFP section profiles 3 dpi, n = 30 vs 26; 10 dpi, n = 40 vs 40; 21 dpi, n = 30 vs 38. (**G**) Control vs IR CVP taste bud profiles 3 dpi, n = 350 vs 244; 10 dpi, n = 530 vs 505; 21 dpi, n = 525 vs 495. (**H**) Control vs IR trench profiles 3 dpi, n = 30 vs 24; 10 dpi, n = 48 vs 48; 21 dpi, n = 43 vs 46.

Previously, we treated mice with a single 8 Gy IR dose, and assessed taste homeostasis in the CVP at progressive days post-IR (dpi)^17^. Here we wanted to compare the impact of repeated IR to that of single dose assessed at comparable time points as well as determine its effect on taste bud renewal in both the fungiform taste papillae (FFP) of the anterior tongue (AT) and the circumvallate taste papilla (CVP) in the posterior tongue. The head and neck of wild-type mice were repeatedly irradiated (4 Gy daily for 5 consecutive days)^35,36^ and tongues harvested during and at progressive time points following IR. Cryosections were immunolabelled with antiserum against KCNQ1, a general taste cell marker^37^, in order to tally and quantify the size of taste bud profiles in irradiated and control tissue. While both FFP and CVP buds were impacted, the manner in which they were affected differed. CVP taste buds of irradiated mice were smaller than controls at 3 and 10 but not 21 dpi (Fig. 1G,I–L), while irradiated FFP taste buds were not significantly smaller at any time (Fig. 1A,C–F). Instead, FFP taste bud number was reduced over time in irradiated mice (Fig. 1B; 10 and 21 dpi). Because taste buds were smaller in irradiated CVP taste buds at some post-IR times, counts required Abercrombie correction (methods and reference^38^, Supplementary Fig. S1), revealing that CVP taste bud number was reduced only at 21 dpi (Fig. 1H,L).

### Fewer differentiated taste cells populate taste buds of the CVP and FFP after fractionated IR

**Figure 2:**
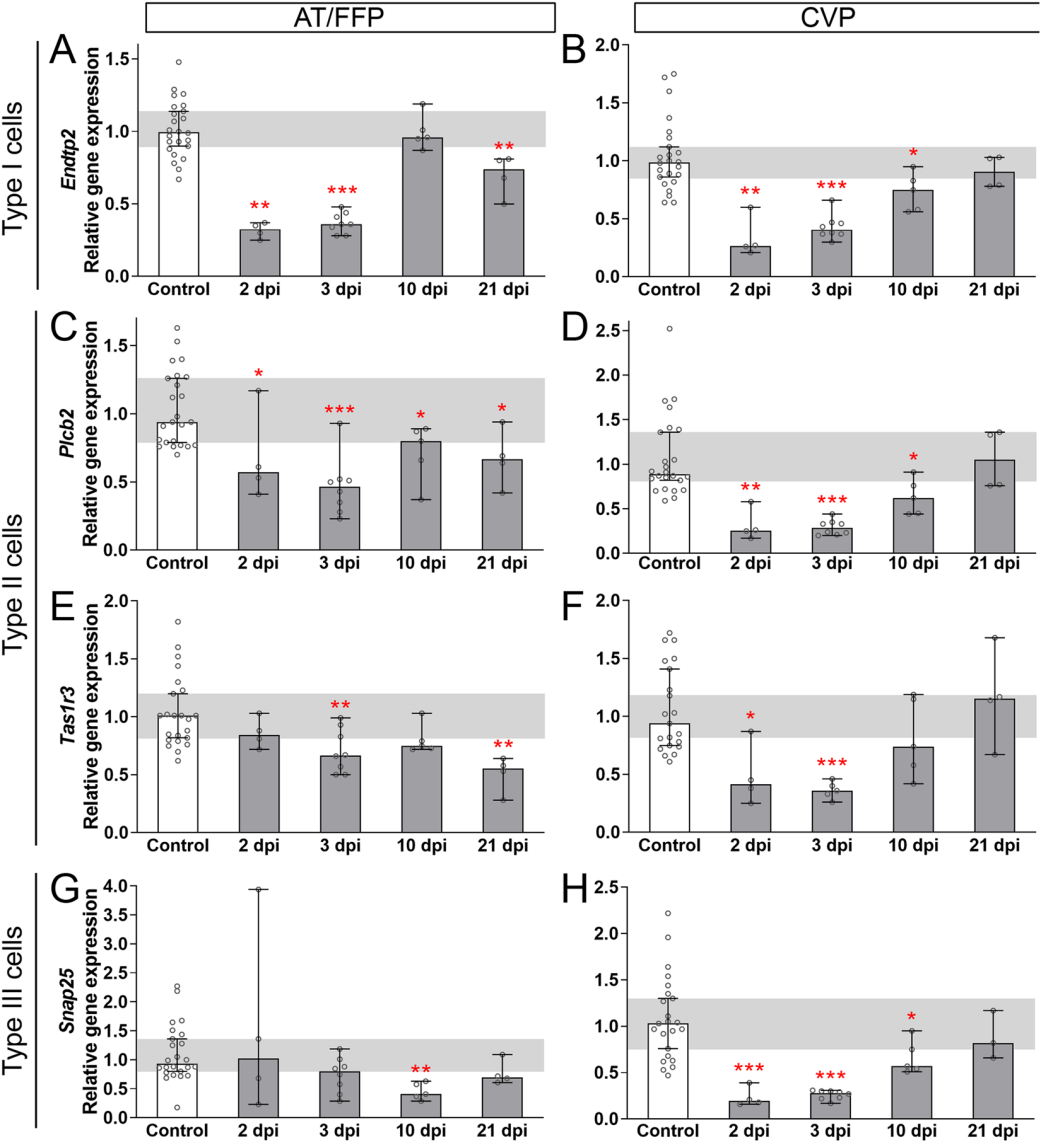
Markers of all 3 differentiated taste cell types are transiently diminished after fractionated irradiation. Expression of markers of all 3 differentiated taste cell types, *Entpd2* for type I, *Plcb2* and *Tas1r3* for type II, and *Snap25* for type III cells was quantified by qRT-PCR. In AT, *Entpd2* expression was reduced shortly after IR, transiently recovered, and then was secondarily reduced at 21 dpi (**A**). *Plcb2* (**C**) and *Tas1r3* (**E**) were significantly reduced at 2–3 dpi, with some recovery, but secondary reduction was observed at 21 dpi. (**G**) In AT, *Snap25* was reduced transiently at 10 dpi. In CVP, *Entpd2, PLCb2, Tas1r3* and *Snap25* were all reduced following IR but recovered by 21 dpi (**B**,**D,F,H**). Data are represented as median (vertical bars) with 95% confidence interval (error bars), and individual points (scatter plot). Horizontal grey bars represent control 95% confidence interval. Mann & Whitney test (*p < 0.05, **p < 0.01, ***p < 0.001). (**A**–**H**) (Controls vs IR mice) 2 dpi, N = 5 vs 4; 3 dpi, N = 9 vs 8; 10 dpi, N = 6 vs 5; 21 dpi, N = 4 vs 4.

Murine taste buds are collections of ~60 elongate taste receptor cells, belonging to 3 morphological types: type I glial-like cells^39–41^, type II sweet, umami, and bitter/high salt cells^42–47^, and type III sour and salt detector cells^47–54^. Because taste buds are reduced in irradiated FFP and CVP, we sought to determine which taste cell types were affected by fractionated IR. Using qRT-PCR, we found marker gene expression for all 3 taste cell types was reduced by fractionated IR, with the kinetics of the IR response differing slightly between CVP and FFP-containing AT epithelium. **(1)** Type I cells express the ectoATPase, NTPDase2^39^; and expression of *Entpd2*, which encodes NTPDase2, was reduced in AT epithelium following IR (albeit with transient recovery at 10 dpi), while in CVP *Entpd2* was similarly reduced post-IR, but with recovery by 21 dpi (Fig. 2A,B). *Entpd2* expression was unexpectedly again downregulated at 21 dpi in AT, but not CVP (Fig. 2A,B). **(2)** *Plcb2*, a general marker of type II cells^55^, was reduced post-IR in both AT and CVP, but only returned to control levels in the CVP (Fig. 2C,D). Type II cells selective for sweet and umami can be distinguished from bitter detecting type II cells by expression of the *Tas1r3* taste receptor. In both AT and CVP epithelium, *Tas1r3* was reduced post-IR; while *Tas1r3* returned to control levels by 10 dpi in both taste fields, its expression in irradiated AT was again downregulated at 21 dpi (Fig. 2E,F). (**3**) Type III taste cells form presynaptic specializations on afferent nerve fibers, and express markers of synapses including SNAP25^56^. In the CVP *Snap25* was downregulated between 2 and 10 dpi (Fig. 2H), while in the AT *Snap25* was lower only at 10 dpi (Fig. 2G).

**Figure 3:**
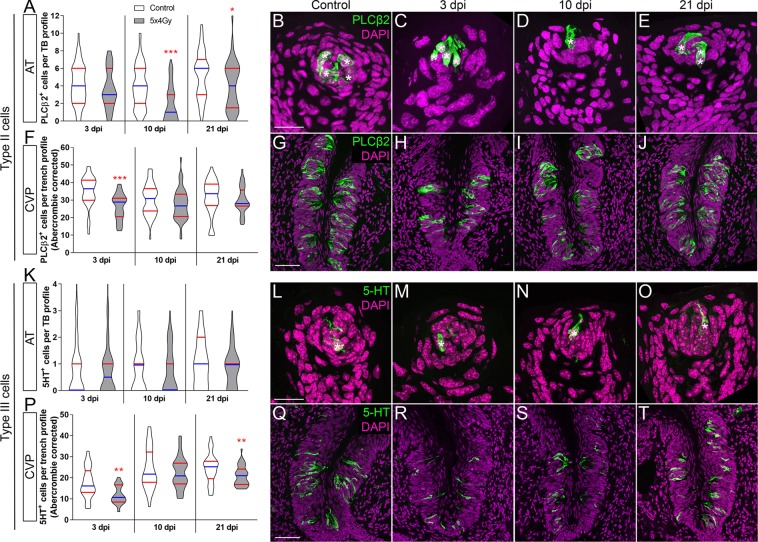
The number of type II and type III cells are differentially diminished after fractionated irradiation, and with different temporal patterns in AT vs. CVP. PLCβ2^+^ type II cell number (green) per taste bud was significantly reduced in FFP at 10 and 21 dpi (**A**–**E**), but only at 3 dpi in the CVP (**F**–**J**). 5-HT^+^ type III cell numbers in FFP were not affected by IR (**K**–**O**). In CVP, 5-HT^+^ cells (green) per trench profile were significantly diminished at 3 and 21 dpi (**P**–**T**). Asterisks indicate PLCβ2^+^ and 5-HT^+^ cells in FFP. Representative pictures are compressed z-stacks. Scale bars 20 µm for (**B–E)**, (**L–O)**, 50 µm for (**G**–**J**,**Q**–**T**). Data are represented as violin plots with median (blue line) and 1^st^ and 3^rd^ quartile (red lines). Mann & Whitney test (*p < 0.05, **p < 0.01, ***p < 0.001). In the FFP, nuclear size of PLCβ2^+^ and 5HT^+^ cells did not differ in control versus irradiated at any time points (Mann & Whitney test, Supplementary Fig. S2A,D), while in the CVP PLCβ2^+^ and 5-HT^+^ cell nuclei were larger in irradiated tongues at 21 and 10 dpi, respectively (Student’s t-test, Mann-Whitney test, respectively. Supplementary Fig. S2B,E); therefore, Abercrombie correction was applied to CVP taste cell counts (see Methods; uncorrected data in Supplementary Fig. S2C,F). (**A**) FFP Controls vs IR mice (N = mice, n = taste bud profiles) 3 dpi N = 4 vs 3, n = 81 vs 59; 10 dpi N = 4 vs 4 n = 83 vs 52; 21 dpi N = 3 vs 4 n = 51 vs 49. (**B**) CVP Controls vs IR (N = mice, n = trench profiles) 3 dpi N = 4 vs 3, n = 31 vs 22; 10 dpi, N = 6 vs 6, n = 48 vs 48; 21 dpi N = 6 vs 7, n = 45 vs 46. (**K**) FFP Controls vs IR (N = mice, n = taste bud profiles) 3 dpi N = 4 vs 3, n = 69 vs 88; 10 dpi N = 4 vs 4, n = 85 vs 53; 21 dpi N = 3 vs 4, n = 57 vs 50. (**P**) CVP Controls vs IR (N = mice, n = trench profiles) 3 dpi N = 4 vs 3, n = 32 vs 18; 10 dpi N = 4 vs 4, n = 30 vs 26; 21 dpi, N = 6 vs 7, n = 32 vs 42.

We next determined if and when the number of specific taste cell types was reduced following fractionated IR. We focused on type II and III cells as these are functional taste receptors for the 5 basic tastes, and can be readily quantified (see reference^57^). In the FFP, the number of PLCβ2^+^ type II cells was significantly reduced at 10 and 21 dpi (Fig. 3A–E); in the CVP, fewer PLCβ2^+^ cells were already evident at 3 dpi, and while trending lower at 10 and 21 dpi did not differ significantly from controls (Fig. 3F–J). Note PLCβ2^+^ cells in the CVP had larger nuclei at 21 dpi and thus these counts required Abercrombie correction (Supplementary Fig. S2A,B, uncorrected data shown in Supplementary Fig. S2C). In addition to expressing SNAP25, Type III taste cells accumulate serotonin (5-HT), detectable with 5-HT antiserum^58^. Fractionated IR did not alter the number of 5-HT^+^ Type III cells in FFP taste buds (Fig. 3K–O). However, 5-HT^+^ cells were significantly fewer in irradiated CVP at 3 and 21 dpi, with a similar trend at 10 dpi (Fig. 3P–T). Note 5-HT^+^ cells in the CVP also had larger nuclei at 10 dpi and thus these counts required Abercrombie correction (Supplementary Fig. S2D,E, uncorrected data shown in Supplementary Fig. S2F). Thus, reduced expression of taste cell marker genes is detectable in both CVP and FFP (Fig. 2) in advance of overt and statistically significant reduction of differentiated type II and III taste cells (Fig. 3).

### Fractionated IR affects progenitor proliferation and causes a reduction in progenitor cell number

**Figure 4:**
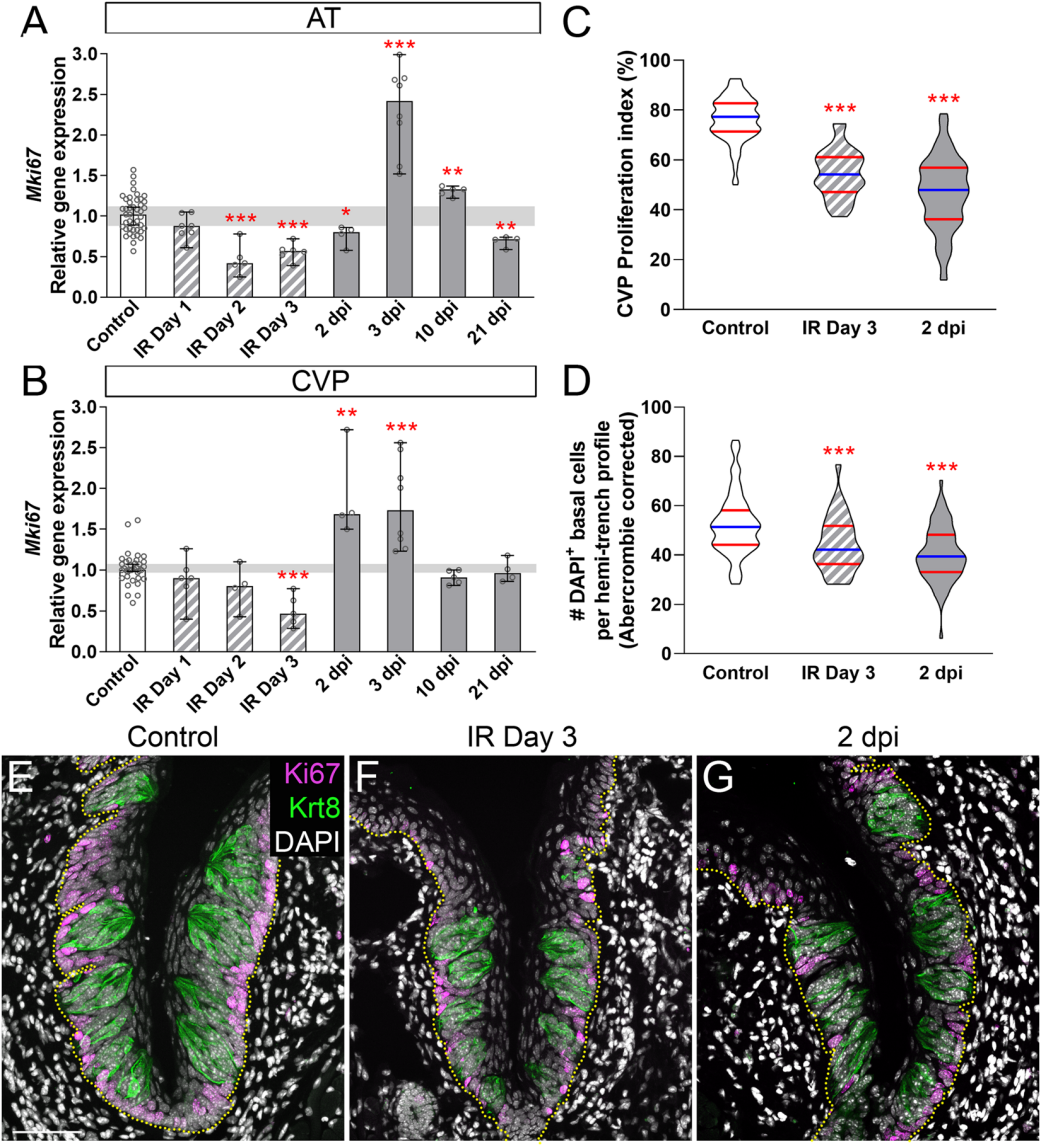
Fractionated irradiation reduces proliferation and causes an overall reduction in basal progenitor cell number. (**A**) In AT, *Mki67* expression was downregulated during (striped bars) (IR Day 2 and 3) and shortly after (gray bars) (2 dpi) fractionated IR, then substantially overshot controls (white bar) before a second drop in expression at 21 dpi. (**B**) In the CVP, *Mki67* expression was significantly downregulated at IR Day 3, overshot controls at 2–3 dpi, and returned to control levels at 10 dpi. (**C**) In the CVP, fractionated irradiation caused a significant reduction in the Proliferation Index (#Ki67^+^ basal cells/#DAPI^+^ basal cells) and led to significantly fewer total DAPI^+^ progenitor cells along the basement membrane (yellow dotted line) (**D**). The size of basal nuclei in control CVP was significantly different in irradiated CVP at 2 dpi (Mann & Whitney test, Supplementary Fig. S3A), and thus required Abercrombie correction (see Methods, uncorrected data in Supplementary Fig. S3B). **(E**–**G**) Representative images are compressed z-stacks of CVP sections immunostained for Ki67 (magenta), Krt8 (taste buds; green), and DAPI counterstained (white). Scale bar 50 µm for (**E**–**G**). (**A**,**B**) Data are represented as median (bars) with 95% confidence interval (error bars), and individual points (scatter plot). Horizontal grey bars represent control 95% confidence interval. Mann & Whitney test (* p < 0.05, **p < 0.01, ***p < 0.001). N AT (Controls vs IR mice) Day 1, 6 vs 7; Day 2, 5 vs 5, Day 3, 8 vs 6; 2 dpi, 5 vs 4; 3 dpi, 9 vs 8; 10 dpi, 6 vs 5; 21 dpi, 4 vs 4. N CVP (Controls vs IR mice) IR Day 1, 5 vs 6; IR Day 2, 4 vs 4; IR Day 3, 3 vs 5; 2 dpi, 5 vs 4; 3 dpi, 9 vs 8; 10 dpi, 6 vs 5; 21 dpi, 4 vs 4. (**C,D**) Data are represented as violin plots with median (blue line) and 1^st^ and 3^rd^ quartile (red line). Mann & Whitney test (*p < 0.05, **p < 0.01, ***p < 0.001). Controls vs IR (N = mice, n = trench profiles) IR Day 3 N = 6 vs 5, n = 73 vs 33; 2 dpi N = 4 vs 5, n = 50 vs 70.

Following single dose IR, progenitor cell proliferation in the CVP is significantly reduced, interrupting production of new taste cells, and thereby causing a transient reduction in type II and III cells as aging taste cells are lost and not immediately replaced^17^. Here, we assessed progenitor proliferation in both FFP and CVP following fractionated IR via qRT-PCR for *Mki67*^59,60^ (Fig. 4A,B). The impact of fractionated IR on *Mki67* expression was roughly comparable in AT and CVP epithelium. In general, and consistent with the impact of IR on many renewing epithelia^17,35,61–65^ *Mki67* was downregulated during IR and overshot controls in the 2–3 days post-IR, before returning to levels more closely aligned with those of controls (Fig. 4A,B). Interestingly, *Mki67* expression in the AT remained elevated at 10 dpi but exhibited a second downregulation at 21 dpi; a secondary reduction in *Mki67* transcripts was not evident in the CVP (Fig. 4A,B).

We also quantified the proportion of Ki67^+^ progenitor cells in immunostained sections of CVP tissue at IR Day 3 and 2 dpi when *Mki67* was reduced and upregulated, respectively (see Fig. 4B). Ki67^+^ and total (DAPI^+^/Krt8^−^) basal cells along the basement membrane (marked with E-cadherin – not shown, outlined with yellow dotted line) were tallied to determine both the total number of basal progenitor cells and the proportion engaged in the cell cycle (proliferation index = Ki67^+^ basal cells/DAPI^+^ basal cells * 100^17^). The proportion of Ki67^+^ cells was significantly reduced both during (IR Day 3) and after IR (2 dpi) compared to controls (Fig. 4C,E–G), as was the total number of progenitor cells (Fig. 4D,E–G). Note progenitor nuclei were significantly larger at 2 dpi (Supplementary Fig. S3A) necessitating Abercrombie correction (Uncorrected data shown in Supplementary Fig. S3B). However, the proliferation index and *Mki67* expression were inversely correlated at 2 dpi (Fig. 4B,C), indicating that proliferative recovery at the transcript level begins in advance of detection of increased Ki67^+^ expressing cells^66^.

Because radiotherapy is known to damage salivary glands, we assessed Ki67 immunostaining in the minor salivary glands associated with the CVP, the Von Ebner’s glands (VEG). While proliferation is robust in control glands, there were many fewer Ki67^+^ cells in irradiated VEG at IR Day 3. Intriguingly, Ki67^+^ cells appeared to rebound at 2 dpi (Supplementary Fig. S3C–H, yellow arrows).

**Figure 5:**
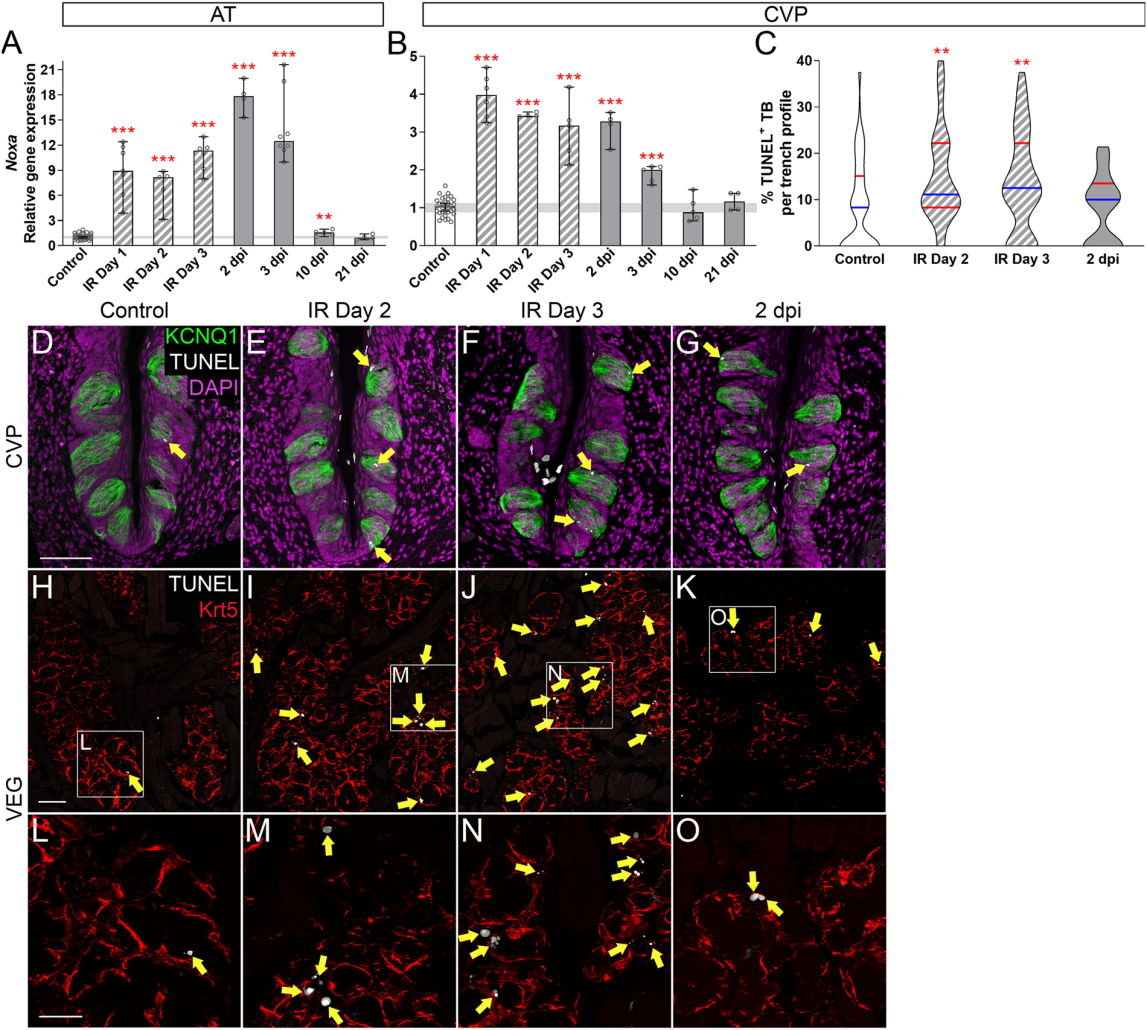
Differentiated cells, but not progenitor cells undergo cell death during fractionated irradiation. (**A**,**B**) In AT and CVP, *Noxa* expression was highly upregulated during (striped bars) and after (gray bars) fractionated irradiation, before returning to control levels at 21 dpi and 10 dpi, respectively. (**C**,**D**–**G**) Quantification of TUNEL^+^ cells in CVP sections immunostained for Krt5 to mark basal progenitors (DAPI magenta and not shown) and KCNQ1 to mark taste buds (green) revealed that TUNEL^+^ cells (white; yellow arrows) were encountered only in CVP taste buds, and not in basal cells (see text). The proportion of TUNEL^+^ taste buds per CVP trench profile was significantly increased during fractionated irradiation (striped violins), and returned to control levels at 2 dpi. (**H**–**O**) TUNEL^+^ cells were also substantially increased in the Von Ebner’s glands (white, yellow arrows; Krt5 red) during IR compared to controls, and appeared to recover at 2 dpi (**H**,**K,L,O**). Representative pictures are compressed z-stacks. Scale bars 50 µm in (**D**) applies to (**E–H**) applies to (**I**–**K**), and 25 µm in (**L**) applies to (**M**–**O**). (**A**,**B**) Data are represented as median (bars) with 95% confidence interval (error bars), and individual points (scatter plot). Horizontal grey bars represent control 95% confidence interval. Mann & Whitney test (*p < 0.05, **p < 0.01, ***p < 0.001). (**A**) AT (Controls vs IR mice) IR Day 1 N = 6 vs 7; IR Day 2 N = 5 vs 5, IR Day 3 N = 8 vs 6; 2 dpi N = 5 vs 4; 3 dpi N = 9 vs 8; 10 dpi N = 6 vs 5; 21 dpi N = 4 vs 4. (**B**) CVP (Controls vs IR mice) IR Day 1 N = 5 vs 6; IR Day 2 N = 4 vs 4; IR Day 3 N = 3 vs 5; 2 dpi N = 5 vs 4; 3 dpi N = 9 vs 8; 10 dpi N = 6 vs 5; 21 dpi N = 4 vs 4. (**C**). Data are represented as violin plots with median (blue line) and 1^st^ and 3^rd^ quartile (red lines). Mann & Whitney test (*p < 0.05, **p < 0.01, ***p < 0.001). Controls vs IR (N = mice, n = trench profiles) IR Day 2 N = 3 vs 3, n = 22 vs 27; IR Day 3 N = 6 vs 5, n = 48 vs 39; 2 dpi N = 3 vs 3, n = 22 vs 21.

Progenitor proliferation is reduced following single dose irradiation, due in part to induction of progenitor cell death^17^. To test here if progenitor death likewise increases following fractionated IR, *Noxa* transcripts (*Pmaip1*, marker of p53-mediated cell death^67^) in AT and CVP epithelia were quantified via qRT-PCR. In both taste fields, *Noxa* was highly upregulated immediately at IR Day 1 and remained elevated during and post-IR before returning to control levels at 21 and 10 dpi in AT and CVP, respectively (Fig. 5A,B). We found previously that after single dose IR, progenitor cells made up the majority of dying cells in the CVP, which was maximal at 18 hours post-IR, with no evidence of taste cell death^17^. Surprisingly, fractionated IR did not induce discernible progenitor cell death. Using Keratin (Krt) 5 immunostaining to label basal progenitors^15,16^, we found that the number of TUNEL^+^/Krt5^+^ progenitor cells per trench profile was extremely low and did not differ between controls and irradiated CVP during or following IR (Mann and Whitney test, IR day 2 p = 0.389, IR day 3 p = 0.277, 2 dpi p = 0.142, respectively; no TUNEL^+^ progenitors per CVP of 3–6 control mice versus 1–2 TUNEL^+^ progenitors per CVP trench of 3–5 irradiated mice). Cell death was also assessed via expression of Cleaved Caspase3, a ubiquitous marker of apoptotic cell death^68,69^. Consistent with the TUNEL data, Caspase3^+^ progenitor cells were rare, and their numbers did not differ between controls and irradiated CVP (Mann and Whitney test, IR Day 2 p = 1, IR Day 3 p = 1, 2 dpi p = 0.232; 0 Caspase3^+^ progenitors per CVP of 3–6 control mice versus 1 Caspase3^+^ progenitor per CVP trench of 3–5 irradiated mice).

Instead, upon fractionated IR, we observed a significant increase in TUNEL^+^ cells inside of taste buds during (IR Days 2 and 3), but not following IR (3 dpi; Fig. 5C,D–G yellow arrows). Likewise, fractionated IR was associated with a higher proportion of taste buds with Cleaved Caspase3^+^ cells on IR Day 2, with a trend at IR Day 3 (Mann and Whitney, p = 0.08. Supplementary Fig. S4A,B–E yellow arrows). In sum, these data suggest that progenitor cell death does not contribute to the decline in progenitors and their proliferation; rather repeated IR induces death of cells within taste buds.

In the AT, where *Noxa* expression was also highly upregulated during and following fractionated IR, we likewise observed limited TUNEL^+^ cells in and around FFP taste buds; specifically, the proportion of taste buds with TUNEL^+^ cells was comparable between control and irradiated mice during IR (IR Day 3, Supplementary Fig. S4F), as was the number of TUNEL^+^ progenitor cells residing at the basement membrane one cell diameter from taste buds (Mann and Whitney test, p = 0.315, 3 TUNEL^+^ progenitors per FFP of 3 control mice versus 1 TUNEL^+^ progenitor per FFP of 3 irradiated mice). Further, as AT epithelium analyzed for *Noxa* expression comprises both FFP and non-taste epithelium, we assessed TUNEL^+^ cells in non-taste epithelium of sections of AT. Similarly, fractionated IR triggered some cell death, albeit not significantly (p = 0.071, Supplementary Fig. S4G–I).

We also assessed the extent of radiation-induced cell death in the VEG, the minor salivary glands associated with the CVP, via TUNEL and Cleaved Caspase3 staining. In controls, TUNEL^+^ and Caspase3^+^ cells were extremely sparse (Fig. 5H,L; Supplementary Fig. S4J,N) but were readily detected in VEG acini especially during IR (Fig. 5I–K,M–O; Supplementary Fig. S4J–Q).

### Wnt/β-catenin signaling is altered in irradiated taste epithelium consistent with a role in recovery of taste cell differentiation but not of progenitor proliferation

**Figure 6:**
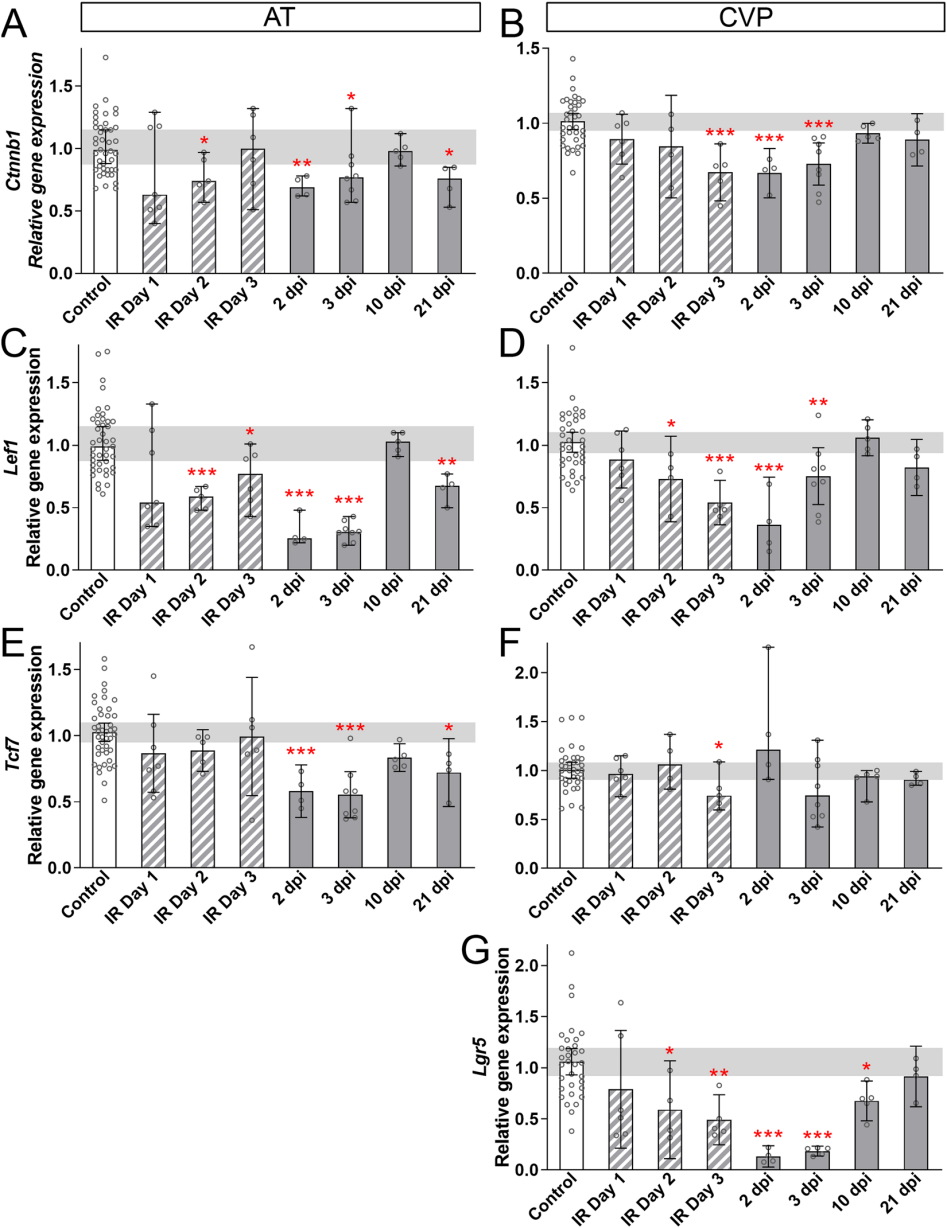
Wnt signaling genes are downregulated during and after irradiation. (**A**,**C**) In AT epithelium, *Ctnnb1* and *Lef1* were reduced during (striped bars) and after (gray bars) fractionated IR, with transient recovery at 10 dpi. (**E**) In AT, *Tcf7* was downregulated but only after IR, with transient recovery at 10 dpi. (**B**,**D**) In CVP epithelium, *Ctnnb1* and *Lef1* were downregulated during fractionated IR (striped bars), and returned to control levels post-IR (gray bars) at 10 dpi with no secondary downregulation at 21 dpi. *Lgr5* is a Wnt target gene expressed by adult taste stem cells in CVP, but not FFP^71^. *Lgr5* expression was similar to *Lef1 in response to IR*, but with delayed recovery (**G**). Data are represented as median (bars) (mean in **B**,**D**,**E**,**G**) with 95% confidence interval (error bars), and individual points (scatter plot). Horizontal grey bars represent control 95% confidence interval. Mann & Whitney test (Student’s t-test in **B,D,E,G**) (*p < 0.05, **p < 0.01, ***p < 0.001). AT and CVP (Controls vs IR N = mice) Day 1, N = 6 vs 7; Day 2, N = 5 vs 5, Day 3, N = 8 vs 6; 2 dpi, N = 5 vs 4; 3 dpi, N = 9 vs 8; 10 dpi, N = 6 vs 5; 21 dpi, N = 4 vs 4.

In taste epithelium, Wnt/β-catenin signaling is required for progenitor survival and proliferation, and regulates taste cell fate decisions prior to differentiation^16,22–24^. Because proliferation of taste progenitors was substantially perturbed by fractionated IR, we explored if, when and how expression levels of β-catenin (*Ctnnb1*), and Wnt target genes *Lef1* and *Tcf7*^23,70^ were impacted. *Ctnnb1* and *Lef1* expression levels dropped during IR, were suppressed for several days post-IR, and then were comparable to controls by 10 dpi in both AT (Fig. 6A,C) and CVP (Fig. 6B,D). While *Tcf7* expression was reduced in both AT and CVP, this reduction was less extensive than for *Lef1* and *Ctnnb1* (Fig. 6E,F); in AT, *Tcf7* was briefly downregulated post-IR (2, 3 dpi), while in CVP *Tcf7* expression was only minimally affected on IR Day 3. In the CVP, *Lgr5* is expressed by taste progenitor cells that give rise to differentiated taste cells^71,72^ and is a mediator, as well as a transcriptional target of the Wnt pathway^73^. *Lgr5* expression mirrored that of *Lef1* in the CVP; it was downregulated during and after irradiation but returned to control levels at 21 dpi (Fig. 6G). *Lgr5* is not expressed by FFP progenitors^74^ but in the AT, we observed a significant secondary decrease in *Ctnnb1*, *Tcf7* and *Lef1* at 21 dpi, reminiscent of secondary reductions at 21 dpi in expression of *Entpd2* (type I taste cells, Fig. 2A), *Plcb2* and *Tas1r3* (type II taste cells Fig. 2C,E), and *Mki67* (proliferation Fig. 4A), suggesting that a longer term perturbation in Wnt pathway activity underlies a secondary decline in FFP taste bud renewal.

## Discussion

Taste dysfunction is an exceedingly common side effect of conventional radiation therapy for head and neck cancers. Here, we developed and characterized a fractionated irradiation protocol in mice to explore mechanisms underlying functional taste loss experienced by patients during and after radiotherapy. We show that, unlike single dose irradiation which leads to progenitor death, fractionated irradiation triggered death of cells within taste buds and did not affect survival of progenitors. However, similarly to single dose IR, repeated irradiation represses proliferation and leads to fewer progenitors. Consequently, fewer new type II and III taste receptor cells are produced. This reduction in new cell supply, combined with death of taste cells leads to smaller and fewer taste buds following IR. Fractionated irradiation also triggers cell death and reduces proliferation in the minor Von Ebner’s salivary glands associated with the CVP. Interestingly, we find that onset of reduced *Mki67* expression is associated with Wnt/β-catenin downregulation, suggesting that Wnt/β-catenin signaling is important to maintain proliferation during radiation exposure. However, following fractionated irradiation proliferation recovers in advance of upregulation of Wnt signaling, suggesting other signaling pathways must govern proliferative recovery. Finally, Wnt signaling levels are restored prior to upregulation of markers of differentiated taste cells, suggesting Wnt signaling may support reestablishment of taste cell differentiation following radiation injury.

### The impact of IR on taste bud homeostasis is more prolonged in FFP compared to CVP

Our data suggest the impact of fractionated IR on taste bud renewal persists longer in the anterior FFP than the posteriorly located CVP. While by most measures CVP taste buds recovered within 21 days after fractionated IR, the AT exhibited secondary reductions in taste cells and taste cell gene expression, as well as *Mki67* expression, suggesting that repeated radiation exposure may lead to long-term disruption of FFP taste cell homeostasis. This effect may be due to a relatively larger amount of radiation delivered to the AT than CVP in our experimental paradigm, as the CVP is located deep in the oral cavity and surrounded by more teeth and bone that can absorb radiation due to their higher density^75,76^. However, data from other cancer therapies as well as genetic studies suggest that FFP homeostasis may be intrinsically more prone to perturbation. In mice, for example, systemic administration of cyclophosphamide, used in the treatment of many cancers^77^ and the cause of substantial dysgeusia for patients^78,79^, has a greater negative impact on FFP taste buds compared to CVP^80^. Likewise, Sonidegib, a Hedgehog pathway inhibitor used to treat basal cell carcinoma patients that also causes significant taste disturbance^81–83^, leads to taste bud loss in both FFP and CVP in mice^84^. However following cessation of Sonidegib, CVP taste buds regenerate in normal numbers, while only partial recovery of FFP taste buds is observed up to 9 months later^84^. Finally, in a genetic model, we found that FFP taste cell renewal is more sensitive to loss of β-catenin than that of CVP^23^. What might account for this differential sensitivity?

Differential gene expression in the CVP compared with the FFP may contribute to the resilience of the CVP to radiation. For example, Lgr5 is expressed by taste progenitor cells in the CVP but not by FFP progenitors^74^. Interestingly, Lgr5^+^ cells in the intestine are important for recovery following irradiation^85^. Therefore, although *Lgr5* expression is greatly downregulated following irradiation in the CVP, residual *Lgr5* expression may be sufficient to mitigate the effect of radiation on the CVP compared to the FFP that do not express *Lgr5*.

The CVP is intimately connected with the Von Ebner’s glands (VEG) that release their contents into the CVP trenches^86,87^. FFP taste buds, by contrast, are bathed in saliva secreted into the oral cavity^88,89^. Hence, the specialized VEG may secrete factors that protect CVP taste bud homeostasis^86,90–93^. Substantial evidence indicates VEG secretions can modulate taste responses^90,94^. Additionally, the VEG expresses EGF that promotes CVP proliferation and differentiation *in vitro*, and TGFα that may protect CVP taste buds from insult^95^. However, salivary glands are also prone to injury by a range of cancer therapies^96,97^. Notably, head and neck cancer patients treated with targeted IR regularly suffer from persistent xerostomia (dry mouth)^98,99^. In mouse models of head and neck IR, irradiated major salivary glands exhibit substantially increased cell death^61,62,100^, have immediately lowered proliferative capacity^61–63^, and persistently reduced salivary function^36,61,101^. Here, we find the Von Ebner’s glands associated with CVP also have increased cell death and diminished proliferation following IR but may be able to engage in proliferative recovery following IR (see Supplementary Fig. S3E,H). This level of damage therefore may not be sufficient to eliminate putative VEG support of CVP taste homeostasis. Thus, it remains an open question as to the nature of the differential resilience of FFP and CVP to radiation and other types of injury.

### The impact of fractionated IR on taste bud homeostasis differs from that of single high dose IR

In our initial study we selected a single dose of 8 Gy to assess the cellular dynamics of taste cell renewal in response to radiation injury^17^. In contrast to the higher doses employed in many studies of mouse lingual epithelium (>13 Gy^31,102–107^), we found this moderate dose was not lethal nor did it induce oral blistering; instead this intermediate IR treatment caused a dramatic reduction in progenitor proliferation, leading to reduced input of new cells into buds^17^. For the current study, we tested several fractionated IR paradigms and selected 5 daily fractions of 4 Gy, for a total dose of 20 Gy. This level of IR (but not 5 × 5 Gy for example) did not lead to mouse lethality but did have a similar effect on taste progenitor proliferation. However, despite the much higher total dose, fractionated IR led to a substantially smaller reduction in proliferative index. While a single IR dose reduced the proliferation index to <20% of controls at 2 dpi^17^, 2 dpi after fractionated IR the proliferation index dropped minimally, to ~70% of controls (Fig. 4C). Further, during and following fractionated dosing, we found no evidence of IR-induced progenitor cell death (see Fig. 5C–G), while progenitor death was significantly increased following 1 × 8 Gy. These differences in cell death and proliferative response suggest that different IR protocols elicit distinct injury responses.

*In vitro*, high levels of UV irradiation trigger apoptosis of skin keratinocytes, while moderate UV exposure spares cells from death, and instead directs keratinocytes to differentiate^108^. Similarly in ventral tongue epithelium of mice, repeated IR depletes the progenitor pool and accelerates epithelial differentiation by inducing increased “abortive” divisions, where progenitors divide to produce 2 differentiated daughters^35^. Thus, we conjecture that in taste epithelium, a single higher dose of irradiation triggers progenitor cell death while repeated lower doses that cause damage below the threshold to induce cell death, may instead drive taste progenitors to differentiate. Intriguingly, we detect TUNEL^+^ cells in taste buds during but not after fractionated IR, leading us to hypothesize that these may be new daughter cells derived from damaged progenitors that enter buds but cannot differentiate and/or do not survive. Additionally, lower dose IR may trigger autophagy rather than apoptosis. In a number of cellular models, mild stress induces autophagy that can support cell survival via recycling of damaged organelles^109–111^. Finally, injury-induced autophagy can lead to cellular senescence^112^. Interestingly, a recent study has identified senescence as a major actor in the ulceration response of rat tongue epithelium to high dose irradiation^113^. Thus, the role of senescence in radiation injury and taste homeostasis may prove a fruitful area of investigation.

In contrast to limited evidence of cell death in taste buds and progenitor cells, we observed a substantial increase in dying cells in the minor VEG salivary glands, suggesting that increased apoptosis in the VEG at least partially may account for persistent upregulation of *Noxa* in CVP epithelium harvested from irradiated mice. However, we also observed sustained upregulation of *Noxa* in irradiated AT epithelium via qPCR, with limited evidence of cell death in tissue sections; further this epithelium is not invested with salivary glands. One potential explanation is that although Noxa is a major mediator of p53-mediated apoptosis^67^, it is also involved in promotion of autophagy in cardiomyocytes^114^, suggesting *Noxa* upregulation in CVP and AT by fractionated IR may reflect induction of autophagy, rather than cell death.

### Temporal regulation of Wnt pathway genes may reflect different roles for Wnt signaling in taste regeneration following radiation injury

We have shown that genetic stabilization of the main effector of the Wnt pathway, β-catenin, in Krt5^+^ progenitors activates proliferation and biases the differentiation of precursor cells into type I and type II cells^16^. By contrast, proliferation is greatly diminished upon deletion of β-catenin^23,24^ or the Wnt ligand Wnt10a^24^, which in turn leads to loss of differentiated taste cells and diminished taste perception in mice. Here, we found *Ctnnb1*, *Lef1, Tcf7* and *Lgr5* expression are highly regulated during and after fractionated IR, but expression of each gene is not necessarily tightly linked and may depend on the discrete roles that each may play to maintain taste bud homeostasis. For example, the function of LEF and TCF transcription factors is highly dependent on the tissue type, isoforms, post-translational modifications, and interactions with co-activators and co-repressors (for reviews, see^70,115,116^). In the non-taste filiform papilla of the anterior tongue, LEF1-mediated transcription promotes proliferation and self-renewal of progenitors, while TCF4 mediates differentiation of filiform keratinocytes^24^. Here we find that downregulation of *Lef1* parallels reduced proliferation in both AT and CVP, while both *Lef1* and *Tcf7* upregulation in the AT correlates with recovery of Type I and II cell marker expression. These observations suggest that in AT taste bud homeostasis, LEF1 regulates Wnt target genes that control progenitor proliferation, while LEF1 and TCF7 together may regulate taste cell differentiation. However, the molecular programs by which lingual progenitor daughter cells are directed to a non-taste versus taste cell fate remain undetermined.

Noticeably, in the AT *Tcf7* expression is not as strongly downregulated as is *Lef1*, and *Tcf7* expression is virtually unchanged in the CVP. However, downregulation of *Tcf7* expression in irradiated epithelium may be masked by infiltration of TCF7^+^ immune cells in response to irradiation-induced inflammation^117^. We have shown previously that TCF7, but not LEF1, is highly expressed by lymphocytes in the lamina propria in close proximity to the CVP epithelium of control mice^23^. Therefore, we cannot rule out that radiation-triggered infiltration of the taste epithelium by TCF7^+^ immune cells enhanced *Tcf7* levels.

### Leveraging Wnt/β-catenin signaling to mitigate radiotherapy-induced taste loss

Our data prompt the suggestion that temporally and spatially precise activation of the Wnt/β-catenin pathway could be used to mitigate radiation damage to taste bud homeostasis. Because Wnt signaling is downregulated coincident with reduced proliferation during fractionated IR, treatment with Wnt pathway activators may rescue proliferation, and subsequently the defect in taste cell renewal. Such an approach has been tested in preclinical mouse experiments to protect oral tissues from radiation injury or promote tissue repair. For example, genetic activation of Wnt1 prevents radiation-induced salivary gland functional impairment^28^, R-spondin1, a secreted factor that promotes Wnt pathway activity, can prevent radiation-induced mucositis^31^ and administration of small molecule GSK3 antagonists supports tooth repair^33^. However, broadly activating the Wnt pathway in parallel with radiotherapy for head and neck cancer may prove detrimental as this approach may trigger tumor resistance or growth^118^. Thus, it is essential to adopt a Wnt activation approach that prevents tumor growth in xenograft head and neck irradiation models^31,104^, such as precise topical and transient delivery of a Wnt pathway activator (for instance as performed with GSK antagonists^33^, Smad7^104,119^ and KGF^63,107^). In addition, targeting elements of the Wnt/β-catenin pathway that are expressed more restrictively in taste tissues but not in cancer cells could potentially lower the risk of tumor growth^120^. Finally, identification of other pathways associated with proliferation reduction and recovery during and after fractionated irradiation, respectively, will highlight new potential therapeutic targets to lessen or prevent taste loss in head and neck cancer patients.

## Materials and Methods

### Ethics statement

All animal procedures were performed in an AAALAC-accredited facility in compliance with the Guide for the Care and Use of Laboratory Animals, Animal Welfare Act and Public Health Service Policy, and were approved by the Institutional Animal Care and Use Committee at the University of Colorado Anschutz Medical Campus.

### Animals and procedures

Mice were housed in sterile ventilated cages with *ad libitum* access to irradiated diet and hyperchlorinated reverse osmosis water delivered *via* an automatic watering system. The light cycle was from 6:00 to 20:00. All mice were on a mixed background (FVB, 129 Sv, C57Bl6). Both males and females between 8–12 weeks of age were used.

### Irradiation

Mice were irradiated with 4 Gy daily for 5 consecutive days (IR Day 1–5) between 8:30 and 9:30 am in a Rad Source RS2000 X-ray irradiator (~1.25 Gy/min, 160 kV, 25 mA). Control and irradiated mice were anesthetized with 300 mg/kg body weight Avertin (2,2,2-Tribromoethanol). Control mice were left in their cages, while irradiated mice were placed in a 50 ml conical tube with the tip cut to allow adequate breathing, empty space filled with cotton, and a lead shield wrapped around the tube to cover the body except the head and neck. Irradiated mice were then returned to their cages to recover from anesthesia. Per IACUC regulation, mice losing more than 15% of their initial body weight (prior to IR on Day 1) were removed from the experiments and euthanized.

### Tissue collection

For immunohistochemistry, mice were first anesthetized by i.p. injection of 300 mg/kg body weight Avertin (2,2,2-Tribromoethanol). Ice-cold normal saline was perfused transcardially prior to perfusion with periodate-lysine-paraformaldehyde (PLP) fixative (75 mM L-lysine monochloride, 1.6% formaldehyde, 10 mM sodium periodate)^121,122^. Tongues were then immersed in PLP fixative for 3 h at 4 °C on a rocker and transferred to 20% sucrose in 0.1 M phosphate buffer (PB) overnight at 4 °C under constant gentle rocking. Samples were embedded in O.C.T. Compound (Tissue-Tek 4583, Sakura Finetek, Torrance CA, USA), frozen on dry ice and stored at −80 °C.

For qRT-PCR, mice were euthanized by CO_2_ inhalation followed by cervical dislocation. Tongues were collected and rinsed in ice-cold Normal Tyrode solution (140 mM NaCl, 5 mM KCl, 10 mM HEPES, 4 mM CaCl_2_, 10 mM glucose, 1 mM MgCl_2_, 1 mM sodium pyruvate, pH 7.4). CVP epithelium was harvested by peeling after enzymatic digestion (Dispase II 3 mg/ml + Collagenase II 1 mg/ml in Normal Tyrode solution injected underneath the epithelium^16^) for 31 minutes at room temperature in calcium-free Tyrode. Harvested tissues were immediately placed in a cold tube on dry ice. Samples were stored at −80 °C.

### Immunohistochemistry

Immunostaining followed previously described procedures^16,22^. Frozen 12 µm cryostat sections were collected on Superfrost Plus Slides (Fisher Scientific, Pittsburgh PA, USA). Primary and secondary antisera, amplification systems, and dilutions are listed in Table 1. Immunoreactivity for each antigen was absent when primary antibodies were omitted.

**Table 1:** Primary and secondary antibodies used for immunohistochemistry.

	Primary antibody	Source *Reference #*/*RRID #*	Dilution	Secondary Antibody	Source *Reference #/RRID #*	Dilution
Taste Cell Markers	Rabbit anti-PLCβ2	Santa Cruz *sc-206*/*AB_632197*	1/1000	Alexa Fluor^®^ 488 donkey anti-rabbit IgG	ThermoFisher Scientific *A11008*/*AB_143165*	1/1000
	Rabbit anti-5HT	Immunostar 20080/*AB_572263*	1/500			
Taste Bud Marker	Goat anti-KCNQ1	Santa Cruz *sc-10646*/*AB_2131554*	CVP 1/500 AT:1/250	Alexa Fluor^®^ 488 or 546 donkey anti-goat IgG	ThermoFisher Scientific *A11055*/*AB_2534102* *A11056*/*AB_142628*	1/1000
	Rat anti-Krt8	Developmental Studies Hybridoma Bank *TROMA-I*/*AB_531826*	1/250	Alexa Fluor^®^ 647 goat anti-rat IgG	ThermoFisher Scientific *A11006*/*AB_2534074*	1/1000
Progenitor marker	Rabbit anti-Krt5	Abcam *ab53121*/*AB_869889*	1/500	Alexa Fluor^®^ 647 goat anti-rabbit IgG	ThermoFisher Scientific *A21245*/AB_2535813	1/1000
Basement membrane	Mouse anti-Ecadherin	Developmental Studies Hybridoma Bank *5D3*/*AB_528116*	1/100	Alexa Fluor^®^ 555 goat anti-mouse IgG2a	ThermoFisher Scientific *A21137*/*AB_2535776*	1/1000
Proliferation Marker	Rabbit anti-Ki67	Thermo Scientific *RM-9106-S*/*AB_2341197*	1/200	Goat anti-rabbit IgG biotinylated	Vector Labs *PK-6101*/*AB_2336820*	1/500
				Streptavidin Alexa Fluor^®^ 488 conjugate	ThermoFisher Scientific *S11223*/*AB_2336881*	1/1000
Apoptosis	Rabbit anti-Cleaved Caspase3	Cell Signaling Technology 9664/*AB_2070042*	1/250	Donkey anti-rabbit IgG biotinylated	ThermoFisher Scientific *65-6140*/*AB_2533969*	1/250
				Streptavidin Alexa Fluor^®^ 546 conjugate	ThermoFisher Scientific *S11225*/*AB_2532130*	1/1000

### PLCβ2, 5-HT, KCNQ1

Double immunohistochemistry was performed as previously described^16^. Here, PLCβ2 or 5-HT rabbit antisera were used for double labeling with goat anti-KCNQ1 on PLP fixed sections. For 5-HT staining, 5-Hydroxy-L-tryptophan (Sigma-Aldrich H9772; 80 mg/kg, 6.4 mg/ml in 0.1 M PB, pH 7.4) was injected i.p. 1 h before fixation^58^. Sections were thawed at room temperature, rehydrated in 1X phosphate buffered saline (PBS), incubated for 1.5 h at room temperature in blocking solution (5% normal donkey serum, 1% bovine serum albumin, 0.3% Triton X100 in 1X PBS pH 7.3), and incubated with primary antisera diluted in blocking solution overnight at 4 °C. Sections were thoroughly washed in 1X PBS + 0.1% Triton X100, and incubated with secondary antisera diluted in blocking solution for 1 h at room temperature and protected from light. Sections were counterstained with DAPI diluted 1/60,000 in 0.1 M PB for 3 min at room temperature, and coverslipped with Fluoromount G.

### Ki67/Krt8/Ecadherin, Cleaved Caspase3/KCNQ1

Sections were thawed, rehydrated in 1X PBS, and antigen retrieval performed in 10 mM sodium citrate pH6 + 0.05% tween20 at 95 °C for 15 min. After cooling to room temperature in antigen retrieval solution, sections were incubated in blocking solution (as above) for 1 h at room temperature, and incubated with Ki67, Krt8 and Ecadherin antisera or Cleaved Caspase3 and KCNQ1 antisera diluted in blocking solution overnight at 4 °C. Sections were washed in 1X PBS, and blocking of endogenous avidin/biotin was performed as above. Sections were incubated with anti-rabbit biotin-conjugated antibody diluted in 1X PBS + 0.1% tween20 + 2.5% normal goat serum (donkey serum for Caspase3/KCNQ1 staining) for 1 h at room temperature. Streptavidin-Alexa 488, goat anti-rabbit Alexa 647 and goat anti-mouse IgG2a Alexa 555 (for detection of Ki67, Krt8 and Ecadherin, respectively) or Streptavidin-Alexa 546 and donkey anti-goat Alexa 488 (for detection of Caspase3 and KCNQ1, respectively) were diluted in 1% bovine serum albumin + 0.3% Triton X100 in 1X PBS and then applied to the sections for 1 h at room temperature. Sections were counterstained with DAPI diluted 1/60,000 in 0.1 M PB pH 7.2 for 3 min at room temperature, washed, and coverslipped using Fluoromount G.

### TUNEL

To assess cell death, the *In Situ* Cell Death Detection Kit TMR red (Roche Applied Science, Cat #12156792910) was used. Sections were thawed at room temperature, rehydrated in 1X PBS prior to antigen retrieval in 0.1 M Sodium Citrate pH6 for 15 min at 90 °C. Sections were washed and incubated in permeabilization solution (0.1% Triton X100 in 0.1% Sodium citrate) for 2 min on ice. Slides were washed and incubated in blocking buffer (50 mM Tris-HCl pH = 7.5, 3% BSA, 20% normal donkey serum) 30 min at room temperature. TUNEL reaction was performed according to the manufacturer’s instructions, by mixing 1 volume of Enzyme Solution with 9 volumes of Label Solution and incubating sections 60 min at 37 °C in a humidified chamber. One negative control was included by incubating a slide with Label Solution only, and one positive control was added by incubating a slide with DNase I (10 U/ml in 50 mM Tris-HCl pH 7.5, 1 mg/ml BSA) 20 min at room temperature prior to the TUNEL reaction. Sections were washed, and incubated for 1 h at room temperature in blocking solution (5% normal donkey serum, 1% bovine serum albumin, 0.3% Triton X100 in 1X PBS pH 7.3), and incubated with Krt5 and KCNQ1 primary antisera diluted in blocking solution overnight at 4 °C. Sections were thoroughly washed in 1X PBS + 0.1% Triton X100, and incubated with secondary antisera diluted in blocking solution for 1 h at room temperature. Sections were counterstained with DAPI diluted 1/60,000 in 0.1 M PB for 3 min at room temperature, washed, and slides mounted with Prolong Gold (ThermoFisher Scientific).

### Image acquisition and analysis

Confocal fluorescence images were acquired using a Nikon A1R laser-scanning confocal Ti2 microscope and NIS Elements software driving a Nikon motorized Ti2-E. VEG images were acquired using a Leica TCS SP8 laser-scanning confocal microscope and LAS X software driving a Dmi8 motorized microscope stand. Immunolabeled cells were tallied by analyzing both 0.75 µm optical sections and compressed z-stacks of 14 optical sections. All sections of the CVP, except the first and last sections with incomplete trenches, were analyzed. Anterior tongue was cut as 12 µm serial sections in 6 sets such that sections on each slide were separated by 72 µm. FFP were analyzed in the 2^nd^ through the 12^th^ section, a region representing 648 µm of the anterior tongue starting ~72 µm from the tongue tip.

Proliferative index in the CVP was calculated as previously^16,17^. The number of Ki67^+^ basal cells was divided by the number of DAPI^+^ basal cells along the basement membrane within the portion of the CVP trenches housing taste buds. Taste bud size was measured by using the NIS Elements Analysis software. Each KCNQ1^+^ taste bud profile was outlined with the Polygon selection tool to measure taste bud area (Area in μm^2^) and the Equivalent diameter in µm calculated (${\rm{Eqdiameter}}=\sqrt{4\times {\rm{Area}}/{\rm{\pi }}}$). Taste buds were counted using the Count tool. When the size of a counted structure was significantly different in control versus irradiated mice, Abercrombie correction^38^ was applied ($P=A\frac{M}{L+M}$; P corrected count, A crude count, M section thickness –12 µm, L structure size – Ki67^+^, PLCβ2^+^, or 5-HT^+^ nuclear width, or taste bud Eqdiameter). The size of 20 nuclei, when applicable, of each cell population was measured on five 0.75 µm optical sections using the NIS Elements Analysis software, and averaged. Each correction factor was calculated and applied specifically for each animal.

### Real-Time RT-PCR

Total RNA was extracted using an RNeasy Plus Micro kit (Qiagen, 74034), and RNA quantity measure by Nanodrop (ThermoFisher Scientific). cDNA was synthesized from 400 ng total CVP RNA or 1000 ng AT RNA using iScript cDNA Synthesis Kit (Biorad) according to manufacturer’s protocol. cDNA equivalent of 2.5 ng total RNA, and 200 nM of the forward and reverse primers were mixed with Power SYBR Green PCR Master Mix (Applied Biosystems). Real-Time PCR was run in a StepOnePlus Real-Time thermocycler (Applied Biosystems) and consisted of forty 95 °C/15 s-60 °C/60 s cycles. The comparative ΔΔCt method was used for relative quantification of gene expression^123^. mRNA levels were normalized to *Rpl19* mRNA levels. Expression of a gene of interest in irradiated tissues was relative to its expression in control tissues. Primers sequences are in Table 2.

**Table 2:** Primers sequences used in Real-Time RT-PCR.

Gene name	Refseq	Forward primer (5′-3′)	Reverse primer (5′-3′)	Source
*Ctnnb1*/*β-catenin*	NM_001165902	CCCAGTCCTTCACGCAAGAG	CATCTAGCGTCTCAGGGAACA	^#^
*Entpd2*/*Ntpdase2*	NM_009849.2	GACAAGGAAAATGACACAGGTATCGTGG	GTTCAAGACATTCAACCAGACTC	^124^
*Lef1*	NM_010703	TATGAACAGCGACCCGTACA	CTCGTCGCTGTAGGTGATGA	^*^
*Lgr5*	NM_010195	CAGCGTCTTCACCTCCTACC	CCTTGGGAATGTGTGTCAAA	^*^
*Mki67*/*Ki67*	NM_001081117	CTGCCTCAGATGGCTCAAAGA	GAAGACTTCGGTTCCCTGTAAC	^125^
*Plcb2*/*Plcβ2*	NM_177568.2	GAGCAAATCGCCAAGATGAT	CCTTGTCTGTGGTGACCTTG	^124^
*Pmaip1*/*Noxa*	NM_021451	AGGAAGGAAGTTCCGCCG	AGCGTTTCTCTCATCACATCACA	^126^
*Rpl19*	NM_009078.2	GGTCTGGTTGGATCCCAATG	CCCGGGAATGGACAGTCA	^127^
*Snap25*	NM_011428	ATCCGCAGGGTAACAAATGATG	CGGAGGTTTCCGATGATGC	^#^
*Tcf7*	NM_009331	CAATCTGCTCATGCCCTACC	TAGAGTGGAGAAAGCTGGGG	^*^

^#^https://pga.mgh.harvard.edu/primerbank/index.html.

^*^http://mouseprimerdepot.nci.nih.gov.

### Statistical analyses

were performed with SigmaStat (Systat Software, San Jose, CA, USA) or Graphpad Prism (San Diego, CA, USA). Normal distribution and equal variances between groups were assessed to determine whether to run a parametric or non-parametric test. A Mann-Whitney test or Student’s t-test was run to determine statistical differences established with a minimum confidence interval of 95%. qPCR data were plotted with controls from all time points pooled after assessing that they were not statistically different (ANOVA and ANOVA on ranks for parametric and non-parametric data, respectively). Non-parametric data are represented as medians, 1^st^ and 3^rd^ quartiles with data distribution (violin plot), while parametric data are represented as scatter plots (individual symbols) with means ± SEM.

## Supplementary information

Supplementary Information

## Data Availability

All relevant data are within the paper and its supporting information files.
