# Supplementary material for: Fractionated head and neck irradiation impacts taste progenitors, differentiated taste cells, and Wnt/β-catenin signaling in adult mice
Source: Sci Rep. 2019 Nov 29;9:17934. doi: 10.1038/s41598-019-54216-9 (PMC6884601; doi:10.1038/s41598-019-54216-9)
Supplement: Supplementary file 1 — Supplementary Information [file 41598_2019_54216_MOESM1_ESM.pdf]

Fractionated head and neck irradiation impacts taste progenitors, differentiated taste cells, and Wnt/β-catenin signaling in adult mice

Dany Gaillard, Lauren Shechtman, Sarah E. Millar and Linda A. Barlow

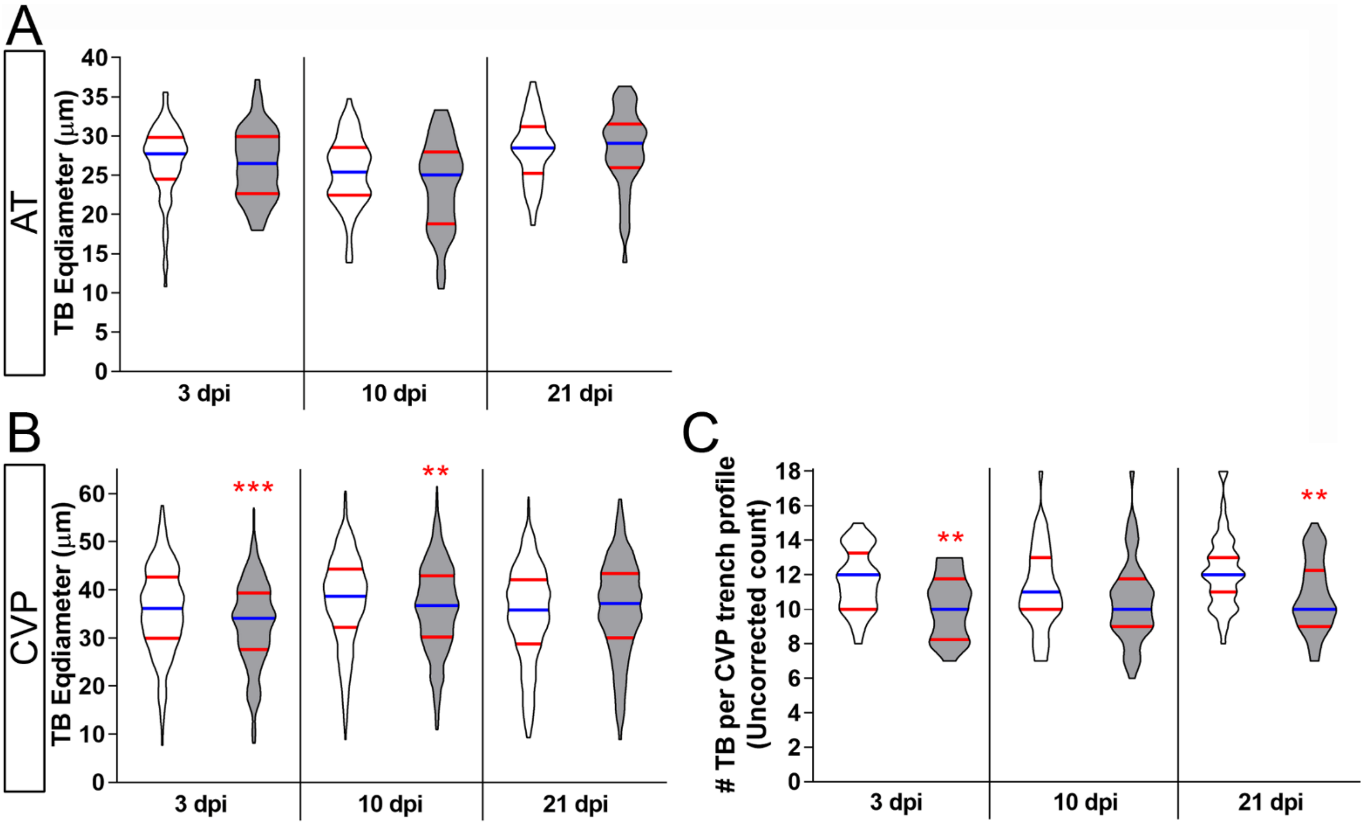

Supplementary Fig. S1. Taste bud Eqdiameter is reduced in CVP, but unchanged in FFP.
(**A**) The Eqdiameter (see Methods) of FFP taste buds in tissue sections did not differ between IR-treated mice and controls. (**B**) Taste bud Eqdiameter was significantly smaller in irradiated CVP at 3 and 10 dpi, and these data were used to apply Abercrombie correction to counts in Fig 1H. (**C**) Uncorrected number of taste buds per CVP trench profile. Data are represented as violin plots with median (blue line) and 1st and 3rd quartile (red lines). Mann & Whitney test (* $p<0.05$, ** $p<0.01$, *** $p<0.001$). (**A**) AT Control vs IR, N=mice. 3 dpi, N=4 vs 3; 10 dpi, N=4 vs 4; 21 dpi N=3 vs 4. (**B**) CVP Control vs IR, N=mice, n= taste bud profiles. 3 dpi, N=4 vs 3, n=350 vs 244; 10 dpi, N=6 vs 6, n=530 vs 505; 21 dpi N=6 vs 7, n= 525 vs 495. (**C**) Control vs IR, n= trench profiles 3 dpi, n=30 vs 24; 10 dpi, n=48 vs 48; 21 dpi, n=43 vs 46.

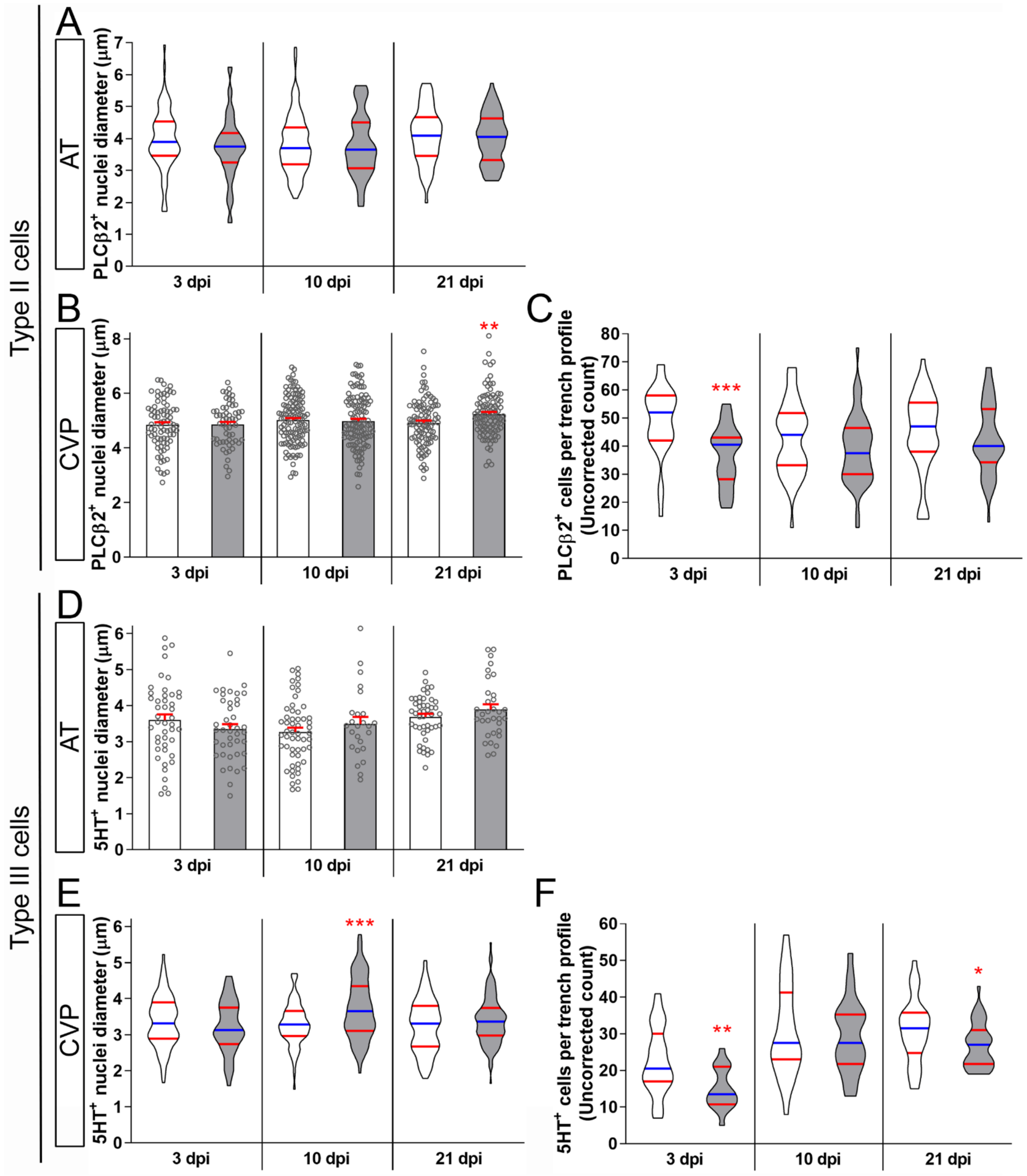

Supplementary Fig. S2. Nuclear size of type II and III taste cells is unchanged in FFP, but is increased in the CVP.

In the FFP, PLCβ2⁺ type II (**A**) and 5-HT⁺ type III (**D**) nuclear size in irradiated tongues did not differ from controls. (**B,E**) In irradiated CVPs, PLCβ2⁺ nuclei were larger compared to controls at 21 dpi, while 5-HT⁺ cell nuclei were larger at 10 dpi (**E**). These data were used to Abercrombie correct taste cell counts in Fig. 3F,P. (**C**,**F**) Uncorrected counts of PLCβ2⁺ and 5-HT⁺ cells per CVP trench profile.

Data are represented as violin plots with median (blue line) and 1st and 3rd quartile (red lines) or mean ± SEM and individual points (scatter plot). Mann & Whitney test in violin plot graphs, Student's t-test in mean ± SEM graphs (* $p<0.05$, ** $p<0.01$, *** $p<0.001$). (**A**) AT Controls vs IR, N=mice, n= nuclear profiles: 3 dpi, N=4 vs 3, n=79 vs 60; 10 dpi, N=4 vs 4, n=79 vs 53; 21 dpi, N=3 vs 4, n=59 vs 81. (**B**) CVP Controls vs IR N=mice, n=nuclear profiles: 3 dpi, N=4 vs 3, n=80 vs 60; 10 dpi, N=6 vs 6, n=120 vs 120; 21 dpi, N=6 vs 7, n=100 vs 101. (**C**) CVP Controls vs IR, N= mice, n=trench profiles: 3 dpi, N=4 vs 3, n=31 vs 22; 10 dpi, N=6 vs 6, n=48 vs 48; 21 dpi, N=6 vs 7, n=45 vs 46. (**D**) AT Controls vs IR, N=mice, n=nuclear profiles: 3 dpi, N=4 vs 3, n=45 vs 41; 10 dpi, N=4 vs 4, n=55 vs 25; 21 dpi, N=3 vs 4, n=47 vs 33. (**E**) CVP Controls vs IR N=mice, n=nuclear profiles: 3 dpi, N=4 vs 3, n=80 vs 60; 10 dpi, N=4 vs 4, n= 80 vs 80; 21 dpi, N=6 vs 7, n=120 vs 140. (**F**) CVP Controls vs IR, N=mice, n=trench profiles: 3 dpi, N=4 vs 3, n=32 vs 18; 10 dpi, N=4 vs 4, n=30 vs 26; 21 dpi, N=6 vs 7, n=32 vs 42.

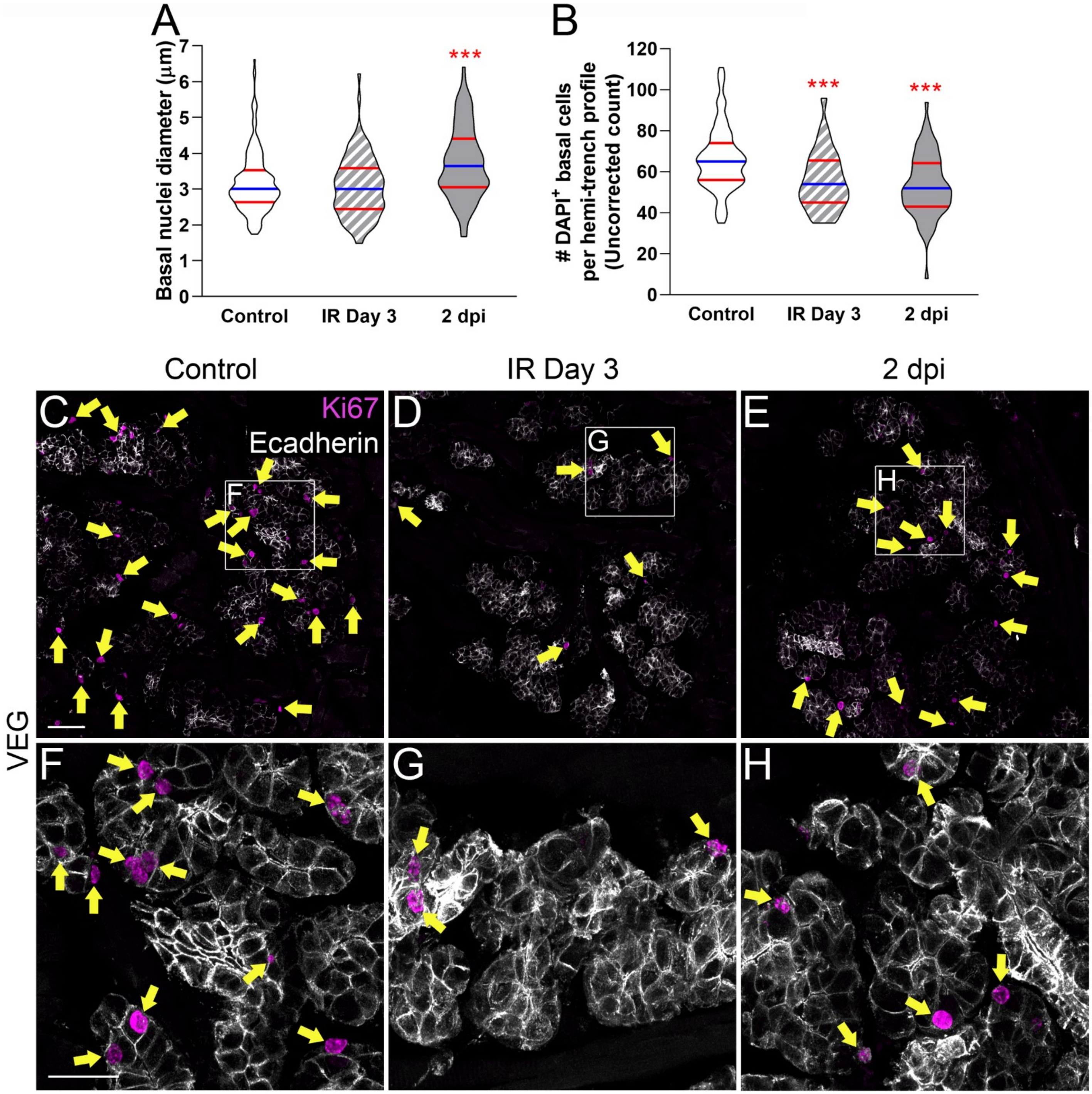

Supplementary Fig. S3. In the CVP, basal cell nuclei are larger after fractionated IR.
Basal nuclei were significantly larger in irradiated CVP compared to controls at 2 dpi (**A**), necessitating Abercrombie correction of nuclear counts in Fig. 4D. (**B**) Uncorrected basal cell counts during and following IR compared to controls. (**C-H**) Ki67^{+} cell number (magenta, yellow arrows) was reduced in Ecadherin^{+} Von Ebner's salivary glands (white) during fractionated IR with some recovery post-IR. (**A,B**) Data are represented as violin plots with median (blue line) and 1st and 3rd quartile (red lines). Mann & Whitney test (*p<0.05, **p<0.01, ***p<0.001). Controls vs IR, N=mice, n=nuclear profiles: Day 3, N=6 vs 5, n=120 vs 100; 2 dpi, N=4 vs 5, n=80 vs 100. (**C,H**) Representative pictures

are compressed z-stacks. Scale bars 50 µm in **C-E**, 25 µm in **F-H**. Controls vs IR mice: IR Day 3, 6 vs 5; 2 dpi, 4 vs 5.

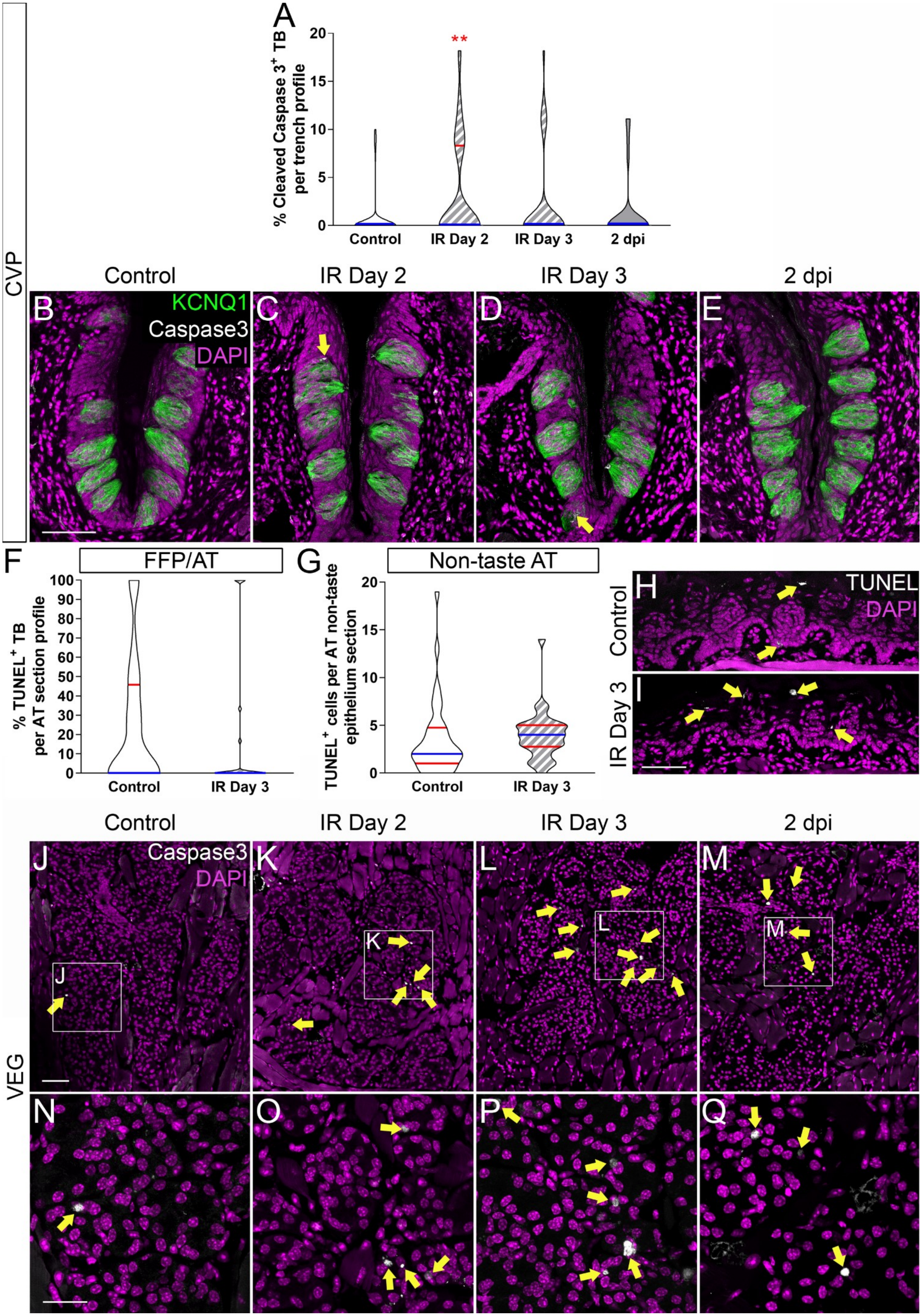
A
% Cleaved Caspase 3+ TB per trench profile
**
Control
IR Day 2
IR Day 3
2 dpi
CVP
B
C
D
E
KCNQ1
Caspase3
DAPI
F
FFP/AT
% TUNEL+ TB per AT section profile
G
Non-taste AT
TUNEL+ cells per AT non-taste epithelium section
H
I
TUNEL
DAPI
Control
IR Day 3
J
K
L
M
N
O
P
Q
VEG

Supplementary Fig. S4. Fractionated IR leads to taste bud cell death in CVP, but not FFP, and to increased cell death in Von Ebner's salivary glands.

(**A**) $Caspase3^+$ taste buds were significantly increased at IR Day 2 and trended upward on IR Day 3 (p=0.08). (**B-E**) $Caspase3^+$ cells (white signal, yellow arrows) were detected in $KCNQ1^+$ taste buds (green) during IR but not post-IR nor in controls (DAPI, magenta). $Caspase3^+$ signal was rarely detected in progenitors in controls or IR treated CVP (see text). (**F**) The proportion of $TUNEL^+$ FFP taste buds did not differ between control and irradiated mice at IR Day 3. (**G**) Fractionated IR increased $TUNEL^+$ cells in AT non-taste epithelium, but not significantly (p=0.071). (**H,I**) Sparse $TUNEL^+$ cells (white signal, yellow arrows) are evident in non-taste epithelium (DAPI, magenta) in controls and IR Day 3. $TUNEL^+$ cells were tallied per 317.44 μm (40x field). (**J-Q**) Cleaved $Caspase3^+$ signal (white, yellow arrows) increased in the Von Ebner's glands during IR and appeared attenuated at 2 dpi. Data are represented as violin plots with median (blue line) and 1st and 3rd quartile (red lines). Mann & Whitney test (*p<0.05, **p<0.01, ***p<0.001). **A.** Controls vs IR, N=mice, n=trench profiles: IR Day 2, N=3 vs 3, n=21 vs 21; IR Day 3, N=6 vs 5, n=42 vs 37; 2 dpi, N=6 vs 4, n=39 vs 27. **F.** Controls vs IR mice: N=3 vs 3 (28 vs 27 AT sections, 65 vs 65 taste buds); **G.** N=3 vs 3 (37 vs 31 non-taste AT sections). Representative pictures are compressed z-stacks. Scale bars 50 μm in **B-E**, 50 μm in **H-M**, 25 μm in **N-Q**.
